# Direct Stimulation of Adult Neural Stem/Progenitor Cells *In Vitro* and Neurogenesis *In Vivo* by Salvianolic Acid B

**DOI:** 10.1371/journal.pone.0035636

**Published:** 2012-04-24

**Authors:** Pengwei Zhuang, Yanjun Zhang, Guangzhi Cui, Yuhong Bian, Mixia Zhang, Jinbao Zhang, Yang Liu, Xinpeng Yang, Adejobi Oluwaniyi Isaiah, Yingxue Lin, Yongbo Jiang

**Affiliations:** 1 Tianjin State Key Laboratory of Modern Chinese Medicine, Key Laboratory of Traditional Chinese Medicine Pharmacology, Chinese Materia Medica College, Tianjin University of Traditional Chinese Medicine, Tianjin, China; 2 Chinese Medical College, Tianjin University of Traditional Chinese Medicine, Tianjin, China; Charité-Universitätsmedizin Berlin, Germany

## Abstract

**Background:**

Small molecules have been shown to modulate the neurogenesis processes. In search for new therapeutic drugs, the herbs used in traditional medicines for neurogenesis are promising candidates.

**Methodology and Principal Findings:**

We selected a total of 45 natural compounds from Traditional Chinese herbal medicines which are extensively used in China to treat stroke clinically, and tested their proliferation-inducing activities on neural stem/progenitor cells (NSPCs). The screening results showed that salvianolic acid B (Sal B) displayed marked effects on the induction of proliferation of NSPCs. We further demonstrated that Sal B promoted NSPCs proliferation in dose- and time-dependent manners. To explore the molecular mechanism, PI3K/Akt, MEK/ERK and Notch signaling pathways were investigated. Cell proliferation assay demonstrated that Ly294002 (PI3K/Akt inhibitor), but neither U0126 (ERK inhibitor) nor DAPT (Notch inhibitor) inhibited the Sal B-induced proliferation of cells. Western Blotting results showed that stimulation of NSPCs with Sal B enhanced the phosphorylation of Akt, and Ly294002 abolished this effect, confirming the role of Akt in Sal B mediated proliferation of NSPCs. Rats exposed to transient cerebral ischemia were treated for 4 weeks with Sal B from the 7th day after stroke. BrdU incorporation assay results showed that exposure Sal B could maintain the proliferation of NSPCs after cerebral ischemia. Morris water maze test showed that delayed post-ischemic treatment with Sal B improved cognitive impairment after stroke in rats.

**Significance:**

Sal B could maintain the NSPCs self-renew and promote proliferation, which was mediated by PI3K/Akt signal pathway. And delayed post-ischemic treatment with Sal B improved cognitive impairment after stroke in rats. These findings suggested that Sal B may act as a potential drug in treatment of brain injury or neurodegenerative diseases.

## Introduction

Ischemic brain damage is one of the most dangerous ailments that lead to learning and memory disability, physical dysfunction and even death. Up to now, no effective treatment has been reported [1]. Neurons as terminally differentiated cells cannot regenerate after injury in traditional view. However, appropriate exercise training can facilitate some neurological function recovery after stroke in clinical practice [2], [3], with the evidence that neurogenesis occurs in the adult brain. Neural stem/precursor cells (NSPCs) had been found and confirmed in adult brain in past decades that it can differentiate into neurons or glial cells as a result of neurogenesis [4]–[7], NSPCs can be stimulated in several pathological conditions, such as neurological diseases, cerebral ischemic in adult brain, and many reports showed that they are an excellent candidate for developing therapeutic strategies to repair the injured CNS [8], [9]. Although the NSPCs would be stimulated to proliferation and differentiation during the brain injury, often this response is not sufficient to overcome the damage. It is essential to study the signalling mechanisms that are activated by small molecular materials in the NSPCs to enhance their response pharmacologically. NSPCs proliferation and neurogensis involves a series of intracellular signaling pathways [10], [11]. Among these pathways, the activation of Notch, mitogen-activated protein kinases (MAPKs) and phosphatidylinositol-3-kinase (PI3K)/Akt pathways are known to play major roles in cell growth and survival responses [12]–[14]. Numerous studies have shown that small molecular materials such as growth factors [15], retinoic acid [16] and Traditional Chinese Medicine (TCM) active constituent [17], [18] can regulate the biological characteristics of neural stem cell and promote neurogenesis. Therefore, regulation of neurogenesis by NSPCs is anticipated as a noble therapeutic strategy for brain damage.

Herbs have been used for treating diseases for centuries, and a lot of natural compounds that with neural beneficial from medicinal plants had been discovered [19]. Treatment of stroke by TCM has a wealth of clinical experience and theoretical basis, and a large number of effective clinical prescriptions have been accumulated. In recent years a large number of studies have shown that TCM prescription and its active ingredient can improve cerebral ischemic injury in experimental animal [20], [21]. Ginsenoside Rb1 and Rg1, for example, improved spatial learning and increase hippocampal synaptophysin level in mice [22]. Curcumin had been demonstrated to stimulate developmental and adult hippocampal neurogenesis, and a biological activity that may enhance neural plasticity and repair [23]. A recent report has shown that NeuroAid (MLC601 and MLC901), a Traditional Chinese Medicine is used in China for patients after stroke, reduced the increase in escape latency and in swim distance induced by ischemia [24]. With an extensive clinical experience, there are ample opportunities to discover natural compounds that effectively promote the proliferation of NSPCs and neurogenesis from TCM.

**Figure 1:**
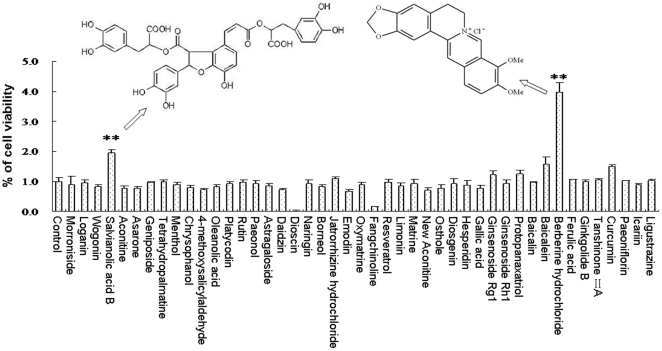
Screening for NSPCs proliferation-inducing natural materials. The proliferation-inducing activities on NSPCs of a total of 45 natural compounds, which were from medicinal materials extensively used in China to treat stroke clinically, were tested using a MTS assay, and the results are expressed in fold change relative to the corresponding controls. The proliferation-inducing effect of the most potent compound, Sal B (A) and berberine (B), were indicated by the arrow and its structure is shown in the inset. Data represent the mean ± S.D. from three independent experiments. **Significant difference from the control group at *P*<0.01.

Sal B was discovered in an *in vitro* screening assay for searching the NSPCs proliferation-inducing natural molecules. It is one of the major ingredients in the water-soluble extracts of *S. miltiorrhiza Bunge* which has been reported to reduce cerebrovascular disease [25]–[27]. As a well-known Chinese herbal medicine, Danshen (*Salvia miltiorrhiza*) has been widely used in traditional Chinese medicinal preparation for the treatment of ischemic disease, and the medicinal properties of this plant have been extensively studied [28], [29]. Several studies have demonstrated the effect of salvianolic acids on preventing brain injury [27], [30]. Importantly, Sal B was reported to be capable of improving the recovery of motor function after cerebral ischemia in rats [31], [32]. However, research on the mechanism of Sal B in treatment of stroke, and the molecular mechanisms responsible for the reported beneficial cerebrovascular effects of Sal B are fairly rare. Meanwhile, the effect of delayed treatment with Sal B after ischemic stroke is still unclear. Therefore, the effect of chronic Sal B treatment beginning seven days after ischemic stroke on neurological deficits and pathophysiology after the transient cerebral ischemic model in rats were studied in our research.

Considering the significance of neurogenesis-related brain recovery, the present study was undertaken to examine the promotive effects of Sal B on NSPCs proliferation and neurogenesis. We provide evidence that Sal B increasing the proliferation of NSPCs in vitro and in vivo. These actions are at least in part mediated by altering the PI3K/Akt signaling pathway. Additionally, delayed post-ischemic administration of Sal B had beneficial effects on the recovery of cognitive function. The stimulative effects of Sal B on NSPCs self-renew and neurogenesis might be associated with a favorable outcome for stroke and other neurological disease.

## Results

### Salvianolic acid B induced the proliferation of cultured NSPCs *in vitro*

Forty-five herbal compounds, which are extensively used clinically for treating stroke in China, were screened in an *in vitro* proliferation assay to identify compounds that could induce proliferation of NSPCs. As shown in Fig. 1, among these natural compounds screened, berberine and Sal B displayed marked activity promoting NSPCs proliferation. In the following study, the proliferative effect of berberine and Sal B were systematically investigated but the action of berberine was proved to be an illusion by BrdU incorporation assay (See Figure S1 in the Supporting Information).

**Figure 2:**
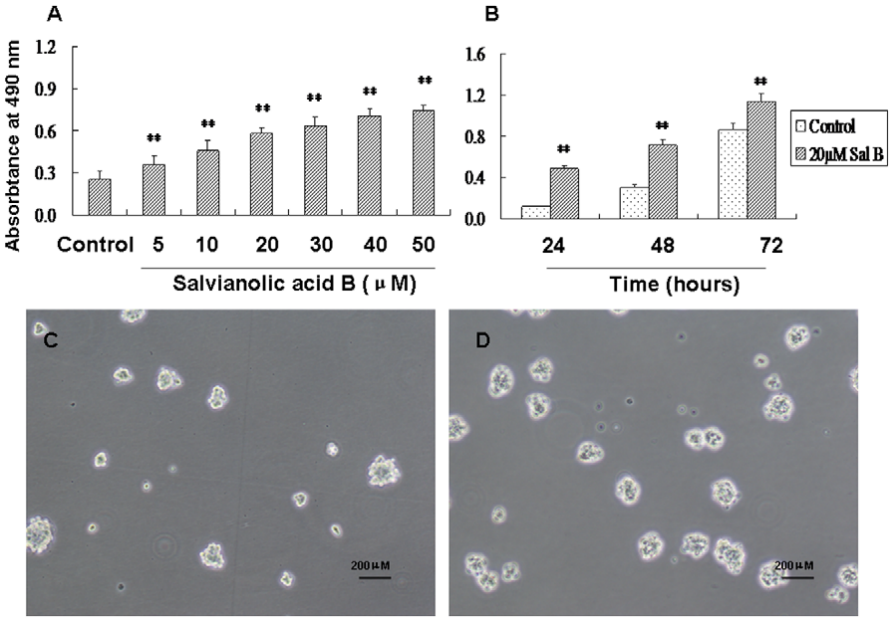
The promotive effect of salvianolic acid B on NSPCs proliferation. (A) Sal B dose-dependently promoted NSPCs proliferation. (B) Sal B time-dependently promoted NSPCs proliferation. Data represent mean ± S.D. from three independent experiments. **Significant difference from the control group at *P*<0.01. (C–D) Representative microphotographs of formed neurospheres in the absence (C) or presence (D) of Sal B (20 µM). Sal B increased the size of formed neurospheres. Scale bars: 200 µm.

To study the proliferation-inducing effect of Sal B in detail, we investigated effects of Sal B on the viability of NSPCs *in vitro* using the MTS assay, NSPCs were treated with Sal B at different concentrations and for different durations. We investigated Sal B at 5, 10, 20, 30, 40, 50 µM dose exposure for 24 hours, and at 20 µM dose incubated for 24, 48, 72 hours on promoting NSPCs proliferation. The results showed that the viability of NSPCs significantly increased as the dose (*P*<0.01, F_(6, 35)_ = 103.06) and time increases (*P*<0.01, Fig. 2A–B). The number and size of neurospheres were increased by addition of 20 µM of Sal B (Figure 2C–D). These results suggested that Sal B significantly increased the viability of NSPCs in dose- and time- dependent manners.

**Figure 3:**
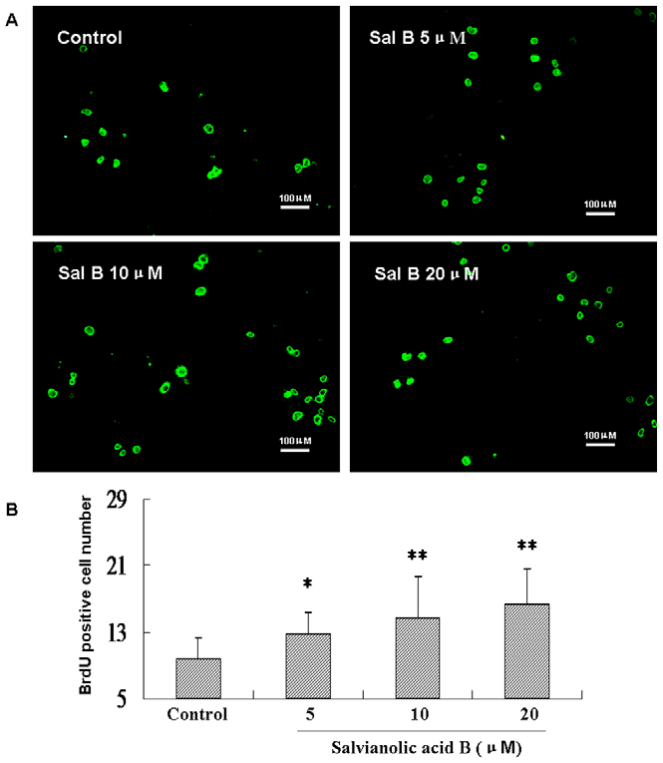
The effect of Salvianolic acid B on BrdU-incorporation in cultured NSPCs. Dissociated NSPCs were cultured on poly-L-lysine-coated 12-well chamber slides with or without Sal B for 12 h and were subsequently incubated with BrdU (10 µg/ml) for other 12 h. After treatment, cells were fixed, immunostained with anti BrdU antibody. (A) Visualization of BrdU-positive cells by the Immunofluorescence staining assay on NSPCs. Scale bar: 100 µm. (B) BrdU-positive cells were counted in 10 randomly selected fields from three different chambers. Data represent the mean ± S.D. from three independent experiments. *Significant difference from the control group at *P*<0.05. **Significant difference from the control group at *P*<0.01.

The MTS assay is a good indicator of cell viability. However, it does not give explicit information on cell proliferation. To provide further evidence that Sal B promotes NSPCs proliferation, a BrdU-incorporation assay was performed. BrdU incorporated cells were detected by immunocytochemistry with anti-BrdU antibody. As shown in Fig. 3, Sal B significantly increased BrdU-labeled cells compared with untreated control (*P*<0.01, F_(3, 40)_ = 6.63, Fig. 3).

### Effect of Salvianolic acid B on self-renew genes of NSPCs

**Figure 4:**
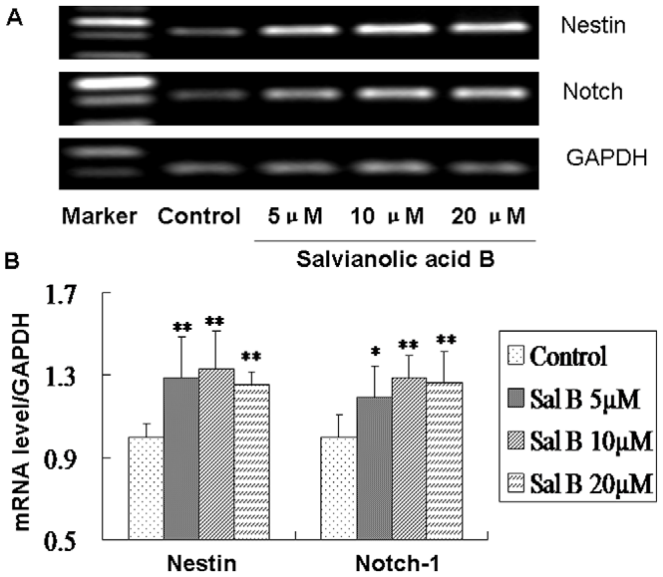
Salvianolic acid B up-regulated the self-renew genes of NSPCs. (A) The mRNA levels of NSPCs self-renew markers were analyzed by RT-PCR. (B) The expression levels were semi-quantified by densitometric measurements, normalized with GAPDH internal control, compared with control, and expressed as means ± S.D from three independent experiments. *Significant difference from the control group at *P*<0.05. **Significant difference from the control group at *P*<0.01.

To confirm the effects of Sal B on maintaining the self-renew cell fate of NSPCs, we further investigated whether treatment of the NSPCs with Sal B could influence their self-renew potential. We studied the effect of Sal B on cell fate specification using RT-PCR method. NSPCs were treated with Sal B (5, 10, 20 µM) for 3 days in proliferation condition, and then the total RNA was extracted. The effect of Sal B on mRNA expression of the self-renew genes, Nestin and Notch-1, was evaluated. The results showed that Sal B up-regulated the expression of Nestin (*P*<0.01, F_(5, 24)_ = 6.92) and Notch-1 (*P*<0.01, F_(5, 24)_ = 6.10) mRNA of NSPCs (Fig. 4). These observations suggested that Sal B can maintain self-renewal of NSPCs.

### NSPCs proliferation mediated by Akt activation

To investigate the molecular mechanism of Sal B on promoting proliferation of NSPCs, we checked the PI3K/Akt, MEK/ERK and Notch signaling pathways, which were closely related to the proliferation and differentiation of NSPCs [12]–[14]. The Akt pathway inhibitor Ly294002, ERK pathway inhibitor U0126, and Notch pathway inhibitor DAPT were used. NSPCs were maintained in the presence or absence of Sal B (20 µg/mL), Ly294002 (20 µM), U0126 (10 µM) and DAPT (10 µM) for 2 days. The proliferative response of Sal B/inhibitors was studied using the cell proliferation assay.

**Figure 5:**
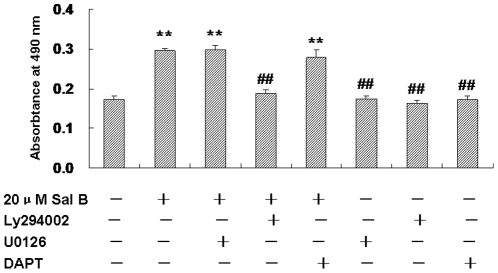
Salvianolic acid B promoted NSPCs proliferation in a PI3K/Akt -independent pathway. NSPCs were cultured in the proliferation medium containing Sal B (20 µM) in the presence and absence of the PI3K inhibitor Ly294002 (20 µM), MEK inhibitor U0126 (10 µM) or Notch inhibitor DAPT (10 µM) for 2 days. Cell survival was assessed by MTS assay. Data represent the mean ± S.D. from three independent experiments. **P<0.01 as compared with control,##*P*<0.01 as compared with Sal B-treated cells.

MTS results showed that Sal B (20 µg/mL) increased the cell survival (Control 0.17±0.01 *vs* Sal 0.30±0.01, n = 5, *P*<0.01). Inhibition of Akt activity by Ly294002 significantly reduced the number of NSPCs, the effects of Sal B on promoting NSPCs proliferation were abolished by the PI3K inhibitor Ly294002, Notch and ERK1/2 had no significant effect on the Sal B -mediated cell survival (*P*<0.01, F(7, 40) = 158.48, Fig. 5). These results suggested that PI3K/Akt pathway is involved in Sal B-induced proliferation of NSPCs.

**Figure 6:**
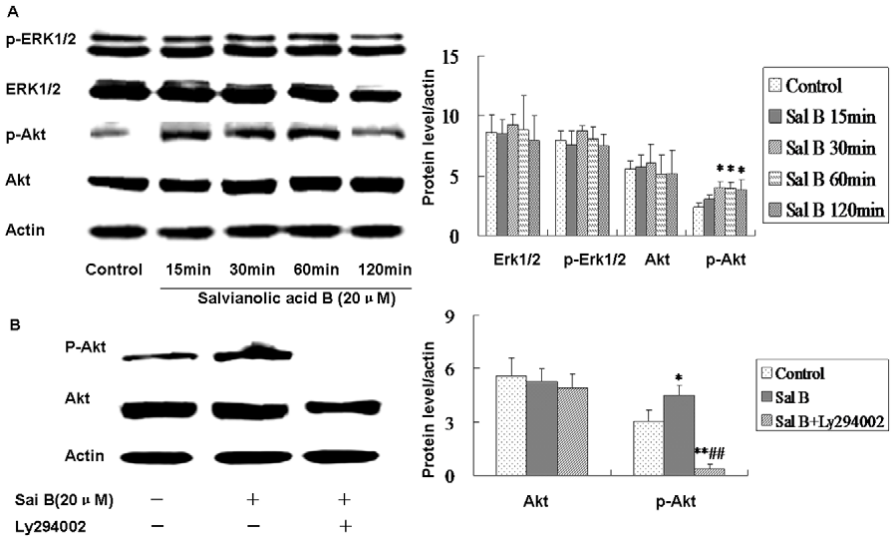
Salvianolic acid B activated PI3K/Akt in NSPCs. Cell lysates from NSPCs treated or untreated with Sal B (20 µM) were subjected to Western blot analysis with antibodies against both total and phosphorylated forms of ERK1/2, and Akt. Actin was used as loading control. (A) Sal B increased the phosphorylation of Akt. Akt phosphorylation in Sal B-treated runners (15 min, 30 min, 60 min, 120 min) were significantly greater than control runners. Histograms represent the change in the phosphorylation of Akt normalized to anti-actin antibodies (n = 4). **Significant difference from the control group at *P*<0.01. (B) PI3K/Akt inhibitors Ly294002 regulated the Sal B-mediated phosphorylation of Akt. Ly294002 abolished the phosphorylation of Akt induced by Sal B. Histograms represent the change in the phosphorylation of Akt normalized to anti-actin antibodies (n = 4). *P<0.05 as compared with control, **P<0.01 as compared with control, ##*P*<0.01 as compared with Sal B-treated cells. No change in total Akt was observed.

We further investigated the phosphorylation of Akt in Sal B-treated cultures. NSPCs were stimulated with Sal B (20 µM) for 15, 30, 60, 120 min and the phosphorylation of Akt was detected using phospho-specific antibodies. Western blotting results showed that Sal B could increase the level of Akt phosphorylation at all of the time tested compared with control (Fig. 6A). The effect of Ly294002 on the blocking of Akt phosphorylation induced by Sal B was investigated by treated NSCPs with Ly294002 (20 µM) and/or Sal B for 30 min. As a result, Ly294002 (PI-3K inhibitor) abolished the phosphorylation of Akt induced by Sal B (Fig. 6B). These results suggested that PI3K/Akt signaling was involved in Sal B mediated proliferation of NSPCs.

### Salvianolic acid B increased the number of BrdU-positive cells *in vivo*

**Figure 7:**
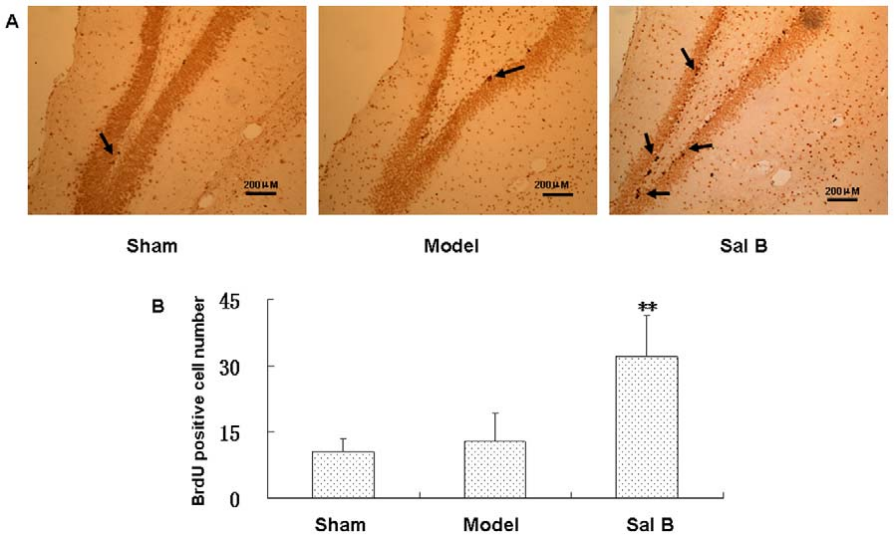
Salvianolic acid B increased the number of BrdU-positive cells *in vivo*. (A) Representative photomicrographs showing BrdU-positive cells in the hippocampus of sham, ischemia and ischemia+Sal B (50 mg/kg)-treated rats. BrdU-labeled cells were indicated by arrows. Scale bar: 200 µm. (B) Quantification of BrdU-positive cells in the hippocampus. Each column represents the represent the mean ± S.D. (n = 10). **Significant difference from the sham group at *P*<0.01.

In the following study, the effect of Sal B on recovery of brain functions after transient cerebral ischemia was evaluated. Firstly, we investigated whether Sal B (50 mg/kg) exposure *in vivo* would affect NSPCs behavior. Since BrdU is incorporated into dividing and proliferating cells, BrdU labeling has been widely used in many investigations concerning neurogenesis. BrdU (50 mg/kg IP) was administered to the rats in sham, vehicle, and ischemia groups once every 2 hours over a period of 8 hours at the 21th day after injection to label the proliferating cells after ischemia. The next day the rats were perfused with 0.01 M PBS (pH 7.2), then with 4% paraformaldehyde (PFA) in PBS. The dividing cells labeled with BrdU were visualized using BrdU immunohistochemistry. BrdU labeled neurons were found in all groups in the dentate gyrus of the hippocampus (Fig. 7). A few BrdU-positive cells were observed in the SGZ of hippocampal dentate gyrus in the control, and BrdU immunoreactivity did not significantly differ between vehicle and sham-operated animals in 21 days. We observed a significant 2.5-fold increase in the numbers of BrdU positive cells in the SGZ of hippocampal dentate gyrus in Sal B treated group (50 mg/kg) (n = 4, *p*<0.01), which is consistent with our *in vitro* findings that Sal B enhanced NSPCs proliferation.

### Salvianolic acid B protected the learning and memory functions

**Figure 8:**
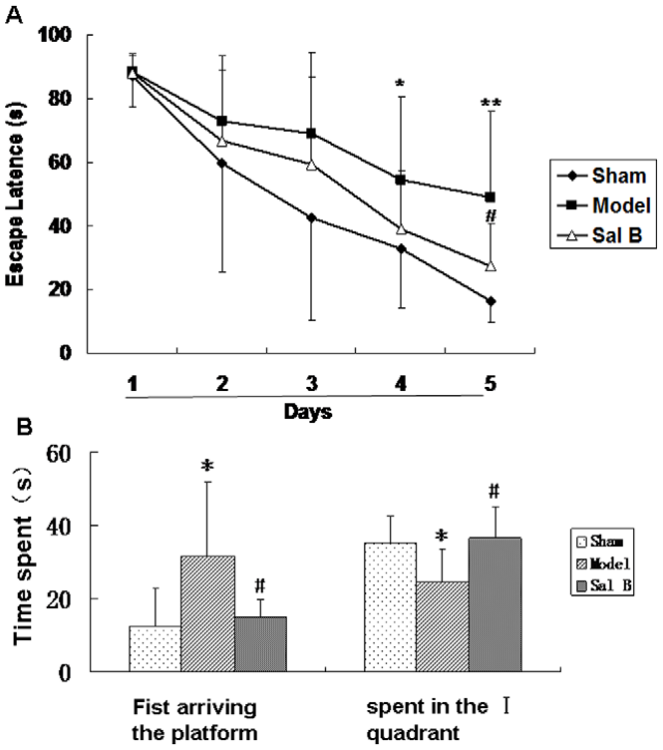
Delayed post-ischemic treatment with salvianolic acid B improved cognitive impairment in Morris water maze task. (A) Place learning with multiple trials. There was a decrease in escape latencies with training in all three groups. (B) In the transfer task, the escape latencies (mean ± S.D.) are compared among sham-operated, untreated ischemic, and Sal B treated groups (n = 8). And the animals from the Sal B-treated group spent more time in the quadrant that contained the escape platform during the place learning than untreated group. *P<0.05 as compared with sham group, #*P*<0.05 as compared with model group.

Since cerebral ischemia can cause prolonged spatial memory disturbance in rats [33], we performed Morris water-maze test to examine the hippocampus involvement and Sal B effect on in learning and memory. Each animal was tested twice a day for 5 consecutive days on their escape latency to the hidden platform. Fig. 8A showed the escape latencies of the three groups of animals during acquisition training (5 days). In all the groups, escape latencies became shorter across training sessions (F_(2,30)_ = 31.54, *P*<0.01, Repeated measures ANCOVA). The decreased escape latency during the learning process in the three groups clearly reflected memory for the escape platform place. Repeated measures ANCOVA revealed no interaction between training days and groups (F_(2,30)_ = 0.90, *P*>0.05). However, differences were observed between the three groups (F_(2,30)_ = 9.96, *P*<0.01, Repeated measures ANCOVA), sham-operated animals memorized the position of the hidden platform very quickly, reaching it in a shorter latency during the training. In contrast, the untreated ischemic animals showed a severe deficiency compared to the control. Although they were gradually trained and showed better performance, their escape latencies were still much longer than those of the control animals. And there are signifitient difference between sham and model of the escape latency in the day 4 (Sham 32.76±18.54 vs Model 54.55±26.05, n = 10, *P*<0.05) and day 5 (Sham 16.38±6.56 vs Model 48.89±27.39, n = 10, *P*<0.01). Administration of Sal B (50 mg/kg) significantly ameliorated such deficiencies.

After 5 days of studying the acquisition of place learning, rats had learned the escape platform position. To determine the degree of memory, on the sixth day the animal was placed in the pool for 90 s without the escape platform and the time the animal spent to arrive the position and in the quadrant which had the escape platform during the training experiment was recorded. Time spent data were shown in Fig. 8. In relation to the transfer of this task, animals in untreated group remained for a similar total period of time in the four quadrants. Animals from sham and Sal B (50 mg/kg) groups spent longer in the quadrant which had contained the escape platform during the initial place learning than in the remaining quadrants. These results showed that Sal B could improve the learning and memory ability of cerebral ischemic animals.

## Discussion

NSPCs proliferation is important to produce of neurogenesis. Compounds that can promote neural stem cell proliferation are seen to support neurogenesis. In the present study, we selected a total of 45 natural compounds from medicinal materials which are extensively used in China to treat stroke clinically, and tested their proliferation-inducing activities on NSPCs using a MTS assay. The screening results showed that several natural compounds such as berberine and Sal B displayed marked effects on the proliferation of NSPCs. In the further research, we found that although berberine could increase the value of MTS assay, it reduced the BrdU incorporation. The action of berberine was proved to be an illusion since BrdU incorporation was considered as an important sign of cell proliferation. Additionally, morphologically berberine could cause cell swelling and decrease the cell number (see Figure S1 in the Supporting Information). Therefore berberine was cytotoxic to neural stem cell and could not promote proliferation.

To explore whether Sal B could promote the NSPCs proliferation, we next systemic examined the promotive effects of Sal B on NSPCs proliferation. Cortical NSPCs were isolated at embryonic day 13.5, the cells were plated in non-adherent conditions, evaluation with MTS assays demonstrated that Sal B induced NSPCs proliferation in a dose- and time-dependent manner. On the molecular level, DNA synthesis of NSPCs in Sal B-treated cultures was determined by BrdU incorporation assay. Immunostaining results showed that the BrdU positive cells were up regulated by Sal B treatment. Consistent with the MTS assay, the BrdU incorporation studies established that metabolic activity of cells was enhanced with the presence of Sal B when compared with the control. These observations suggested that Sal B indeed promoted the proliferation of the NSPCs. Next we conducted a RT-PCR analysis with NSPCs self-renew marker, Nestin and Notch-1. The results showed that the nestin and Notch-1 mRNA expression was up-regulated significantly by Sal B treatment. The results were consisted with the promotion of NSPCs proliferation induced by Sal B. In summary, these observations suggest that Sal B could maintain the NSPCs self-renew and promote proliferation.

Several intricate cell signaling cascades are crucial for determining whether NSPCs proliferation or differentiation. Notch, MEK/ERK and PI3K/Akt pathways are the most frequently ones that associated with regulation of cell growth, survival, and differentiation [34]. We sought to determine which signaling pathway was activated by which Sal B facilitated the NSPCs self-renew. If these signal pathways play important proliferation roles in NSPCs, inhibition of PI3K/Akt should reduce Sal B-induced proliferation. MTS results showed that the absorbance value could be increased by Sal B, and PI3K/Akt specific inhibitor Ly294002 but not Notch specific inhibitor DAPT and MEK/ERK specific inhibitor U0126 could block this effect. The results suggested that PI3K/Akt signal pathway was involved in the Sal B-induced proliferation of NSPCs.

To gain further insight into the mechanisms by which Sal B modulates PI3K/Akt signalling and by which PI3K/Akt mediates Sal B induced self-renew, we evaluated the role of the PI3K/Akt pathway. Akt is a serine/threonine kinase, which can be activated by phosphorylation and subsequently enhance cell survival by activates multiple downstream targets [35], [36]. We demonstrated that exposure to Sal B induced the activation of Akt. If PI3K/Akt signal pathway plays an important prolifeation role, inhibition of PI3K/Akt should reduce Sal B-induced phosphorylation of Akt. Consistent with our hypothesis, the results showed that Sal B-induced activation of Akt was completely inhibited by the PI3K/Akt-inhibitor, Ly294002. In short, these findings supported the hypothesis that PI3K/Akt signal pathway was the signal mediator in Sal B-stimulated NSPCs proliferation.

In order to examine the effects of Sal B on proliferation of NSPCs *in vivo*, transient cerebral ischemic rats and BrdU labeling were employed. New neurons can be generated in the hippocampal of adult rats after transient ischemia of the forebrain [37]–[39]. Many researchers had reported that cerebral ischemic could transient stimulated neurogenesis in the adult hippocampus. As far as we know, Liu et al. [40] first demonstrated increased neurogenesis in the gerbils hippocampus after transient global ischemia, the BrdU incorporation peaks 11 days after ischemia and that incorporation subsides to control levels by 3 weeks after ischemia. Similar results were described after transient global ischemia in mice [41] and rats [42]. But most of the cells were not fully mature during the 2- to 5-week period after ischemia and could not form a functional link [42]. Therefore, in our view, the behavior performance could not be better at that time. In accordance with previous reports, the NSPCs responded to the injury by proliferation in this study. Cerebral ischemic made the NSPCs proliferation after 7 days (See Figure S2 in the Supporting Information), but the proliferation ability of the rats in the vehicle group declined quickly 28 days after cerebral ischemic. However, Sal B (25 mg/kg) treatment significantly increased the number of BrdU-positive cells in the dentate gyrus 21 days after injection, suggested that Sal B exposure could maintain the ability of the NSPCs proliferation *in vivo*.

Since Sal B promote NSPCs proliferation *in vitro* and *in vivo*, we next sought to correlate the regeneration of new neurons and recovery of brain functions. The present study demonstrated that delayed post-ischemic treatment (7 days after ischemic stroke) with Sal B (25 mg/kg) improved cognitive impairment after stroke in rats. Since NSPCs proliferation in hippocampus was important site for spatial learning and memory [43], [44], we speculated that the enhancement of functional recovery by Sal B might be dependent on its action on NSPCs proliferation. But more extensive experiments were still necessary to demonstrate that Sal B could cross over the blood–brain barrier and act on NSPCs. Further study will be carried out to clarify this point. Although our present study showed that Sal B promoted the adult hippocampus neurogenesis and improved the cognitive functions in cerebral ischemia rats, there is no direct evidence to show that Sal B could pass through the blood-brain barrier [45], and the exact mechanism(s) by which Sal B acts on adult neurogenesis remain unclear. More advanced research is needed in the future to further clarify the pathways and mechanisms of Sal B in promoting adult neurogenesis including using appropriate tool drugs to block certain pathways to confirm NSPCs proliferation by Sal B on contributing to the cognitive improvement.

In conclusion, the results of this work clearly demonstrated that Sal B was capable of promoting proliferation of NSPCs and improving the learning and memory ability of cerebral ischemic rats. Additionally, we confirmed that Sal B promoteed NSPCs self-renew and neurogensis were at least in part mediated by the PI3K/Akt signaling pathway. These findings suggested that Sal B may act as a potential drug in treatment brain injury or neurodegenerative disease.

## Materials and Methods

### Reagents

Salvianolic acid B (Sal B, purity >99%) was purchased from the Chinese National Institute for the Control of Pharmaceutical and Biological Products (Beijing, China), When used, it was freshly prepared in phosphate buffer solution (PBS); B27 (without retinoic acid) was purchased from Invitrogen (Carlsbad, CA, USA); Recombinant human fibroblast growth factor 2 (FGF-2) was from Millipore (Temecula, CA); Antibodies for ERK1/2, phospho-ERK1/2, Akt, phosphor-Akt (Ser-473) were obtained from Cell Signaling Technology (Beverly, MA). Antibody for β-actin was purchased from senta; 1,4-diamino-2,3-dicyano-1,4-bis(2-aminophenylthio)-butadiene (U0126) and 2-(4-morpholinyl)-8-phenyl-4H-1-benzopyran-4-one (Ly294002) was from CalBiochem (San Diego, CA). U0126 and Ly294002 were solubilized in dimethylsulphoxide.

### Cell culture

Primary neurospheres were isolated from the cerebral cortex of 13.5-day-embryonic Wistar rats, according to the method described by Davis et al [46]. Briefly, the cerebral cortex was carefully isolated from adjacent tissues, and collected in cold serum-free medium consisting of DMEM/F-12 (1∶1; Invitrogen, Carlsbad, CA, USA). The tissue was digested at 37°C for 10 min by acctuase (Millipore, Temecula, CA), and mechanically disrupted into single cells by filtering through a nylon mesh of 70 µm. The dissociated cells were then plated at a concentration of 2×10^5^ cells/ml in T75 culture flasks for 20 ml, and cultured in neurosphere proliferation media consisting of Dulbecco's Modified Eagle's Medium (DMEM): F12 supplemented with 2% (v/v) B27 supplement (Invitrogen, Carlsbad, CA, USA), 20 ng/ml epidermal growth factor (EGF; PEPROTECH, Rocky Hill, NJ, USA), and 20 ng/ml fibroblast growth factor 2 (FGF2). After 6 days in culture, the proliferating cells formed the neurospheres, which were suspended in the medium. Subsequently, the neurospheres were passaged by treatment with accutase about 5 min at 37°C until they were gently dissociated, and then subcultured as single cells in a new T75 culture flask at a density of 20,000 cells/ml in the fresh culture medium. The procedure of subculture was repeated again to achieve the purified cortical NSPCs and proliferating neurospheres. The 3–5 passages of NSPCs were used for the following experiments.

### MTS assay

For *in vitro* cell proliferation assay, A Cell Titer 96 AQ_ueous_ One Solution Cell Proliferation Assay (Promega, Charbonnières-les-Bains, France) was used. NSPCs were plated at 30, 000 cells/well in a 96-well plate in the presence and absence of Sal B (0.5, 1, 5, 10, 20 µM), U0126 (10 µg/ml), DAPT (10 µg/ml) and Ly294002 (20 µg/ml) in a 96-well plate. Cell proliferation was assessed at day 2 of cell culture. According to the manufacturer's recommendations, 40 µl MTS solution was then added into each of the wells. Then cells were incubated for 2 h at 37°C in the humidified 5% CO_2_ atmosphere incubator, and results were obtained at a wavelength of 490 nm using a microplate reader (FlexStation 3, Molecular Devices, USA). The same volume of medium without cells was used as blank. Results were expressed in Optical Density (OD).

### *In vitro* BrdU-incorporation assay

For *in vitro* BrdU-incorporation assay, cultured NSPCs were incubated with or without Sal B for 24 h, the cells were labeled with BrdU (10 µg/ml) during the last 12 h of incubation, then the cells were plated onto poly-L-lysine coated slides for 2 h, fixed in 4% paraformaldehyde, washed with phosphate-buffered saline (PBS) and incubated in 2 N HCl at 37°C for 10 min. After washing with PBS, the cells were incubated with mouse anti-BrdU antibody (1∶100, senta) at 4°C for 24 h in PBS. After washing in PBS, they were then incubated at room temperature for 1 h in PBS containing FITC conjugated anti-mouse IgG secondary antibody (1∶200, senta). BrdU-positive cells were evaluated using a fluorescent microscope (Leica, German). BrdU positive cells were counted in 10 randomly selected fields from three different chambers.

### RT-PCR analysis

Cells were harvested and total RNA was isolated from treated or untreated NSPCs with TRIzol (Roche), the first strand cDNA was synthesized from 0.4 µg of total RNA using Reverse Transcriptase (Takala) and Random primer as described in the manufacture's instructions. After synthesis, 5 µl of cDNA was used in PCR reaction with gene-specific primers, The sequences of the PCR primer pairs (5′ to 3′) that were used for each gene are as follows: rat nestin, 5′- TTCCCTTCCCCCTTGCCTAATACC-3′ (forward) and 5′- TGGGCTGAGCTGTTTTCTACTTTT-3′ (reverse); Notch-1, 5′- ATGGCCTCCAACGATACTCCT-3′ (forward) and 5′- ACATGTACCCCCATAGTGGCA-3′ (reverse). Products were analyzed on 1.5% agarose gel and visualized by ethidium bromide staining. The relative amount of each transcript was normalized to the level of β-actin.

### Western blot analysis

Protein extracts were prepared and subjected to Western blot analysis. Cells were harvested and lysed with RIPA lysis buffer. The protein concentrations of the lysates were determined with a Bradford protein assay kit (Bio-Rad) according to the manufacturer's instructions. An equal amount of protein was fractionated by SDS-polyacrylamide gel electrophoresis (PAGE) and transferred onto polyvinylidine difluoride membranes. After blocking with 5% skim milk in TBS-T, the membrane was probed with primary antibodies (goat anti-actin 1∶1000, Santa Cruz; rabbit anti-ERK1/2 1∶500, rabbit anti-phospho-ERK1/2 1∶300, rabbit anti-Akt 1∶500 or rabbit anti-phospho-Akt (Ser-473) 1∶300, Cell Signaling Technology) in a blocking solution of non-fat milk (5%). Secondary horseradish peroxidase-conjugated rabbit anti-goat 1∶2000, goat anti-rabbit 1∶2000 (Santa Cruz) antibody in non-fat milk blocking solution (5%) was then applied. The immuno-reactivity was visualized with ECL Western blotting detection reagents (Millipore).

### Induction of the transient global ischemia

Adult (8–10 week-old) male Wistar rats weighing 200–250 g were subjected to transient forebrain ischemia by a method combining those described previously [47], [48]. The rats were housed singly in temperature-controlled conditions with a 12 hr light/dark cycle (lights on: 8:00 AM) following surgery. They had access to food and water ad libitum. Experimental procedures were conducted in accordance with recommendations of the Animal Ethics Committee of Tianjin University of Traditional Chinese Medicine (TCM-2009-034-E01). In brief, twelve hours before the induction of ischemia, rats were anesthetized with sodium pentobarbital, the bilateral carotid arteries were exposed to facilitate the occluding on the following day, and then the vertebral arteries were irreversibly occluded by electrocoagulation. The next day, ischemia was induced by bilaterally occluding carotid arteries with aneurysm clips, and carotid arteries were clamped for 6 min exactly. Rats lost their righting reflex during ischemic, the clips were removed to restore cerebral blood flow. The rats that remain their righting reflex in 1 min after occluding of the both carotid arteries were considered to be failure of ischemia and eliminated. Sham-operated rats were anesthetized, the carotid arteries were isolated, but they were not clamped.

### Drug adminstration

The study was carried out on rats divided into three groups (n = 13). (i) Sham group+vehicle (Sham), (ii) Ischemic group+vehicle (Model) and (iii) Ischemic group+Sal B (Sal B). Seven days after cerebral ischemia, Sal B (50 mg/kg) was dissolved in distilled water, and injected i.p. once daily for 4 weeks, and three mice in each group were used for histological analysis at day 21 after innjection. All controls received an amount of vehicle equivalent to drug treatment conditions.

### *In vivo* proliferation assay

For *in vivo* proliferation analysis of Sal B, that had not been behaviorally tested were evaluated for BrdU labeling. BrdU (50 mg/kg; Sigma) was injected i.p. once every 2 hours over a period of 8 hours at the 21th day after injection to label the proliferating cells. Twenty-four hours after the last BrdU injection, rars were anesthetized with ether and perfused with PBS, followed by a cold 4% paraformaldehyde solution. Brains were collected and post-fixed overnight in a 4% paraformaldehyde solution at 4°C. Coronal sections (20 µm thickness) were obtained throughout the hippocampus. For analysis concerning BrdU immunohistochemistry, sections were incubated in 50% formamide/2×saline sodium citrate (SSC) for 2 h at 65°C, followed by a rinse with PBS. Sections were then incubated in 2 N HCl for 30 min at 37°C to denature double-stranded DNA, and rinsed in 0.1 M borate buffer (pH 8.5). After blocking for 2 h with 1% BSA in PBS, sections were incubated overnight at 4°C with mouse anti-BrdU monoclonal antibody (1∶100; senta). Followed by rinsing in PBS, sections were incubated for 2 h at RT with biotinylated goat anti-mouse IgG (1∶200; senta), and incubated for 2 h at RT with the ABC kit. BrdU positive cells were visualized by incubating sections with Vector DAB.

### Morris water maze task

The learning and memory ability was examined using the Morris water-maze [49]. A cylindrical tank 1.5 m in diameter was filled with water (22±1°C), and a transparent platform 10 cm in diameter was placed at a constant position in the center of one of the four quadrants within the tank. The platform was set 2 cm below the water level where the rats could not see it directly. Rats were allowed to swim freely for 1 min to become habituated to the apparatus. From the next day, in the hidden platform trials, acquisition trials were carried out 2 times per day for 5 days. In each trial, rats were placed into the water at a fixed starting position, and the time taken to escape onto the hidden platform and the swimming path length were measured. Rats were given 90 s to find the hidden platform during each acquisition trial. If it failed to locate the platform within 90 s, it was guided there. The rat was allowed to stay on the platform for 20 s. Performance was tested 24 h after the final training day in a probe trial during which the platform was removed, the rat was placed in the start and its behavior was monitored for 90 s.

### Statistical analysis

SPSS11.5 for Windows (SPSS Inc.) was used to analyze the data. Data are expressed as the mean ± S.D. and analyzed by ANOVA followed by the post-hoc Least Significant Difference (LSD) test. Differences were considered statistically significant if P values were less than 0.05. To analyze water-maze place-navigation performance, the average escape latency of 5 trials per day per animal was calculated and evaluated by repeated-measures ANCOVA.

## Supporting Information

Figure S1 **Berberine failed to promote the NSPCs proliferation.** (A) Berberine increased the value of MTT assay. (B) Berberine reduced the BrdU incorporation. **Significant difference from the control group at *P*<0.01. (C) Morphologically berberine caused cell swelling and decreased the cell number. Scale bar: 200 µm.
(TIF)

Figure S2 **Regeneration of Hippocampal NSPCs Following Ischemia.** BrdU positive cells in the granule cell layer in the intact (A) and ischemic (B, DAI7) animals. The inset shows an enlarged display of a typical BrdU positive cell. Scale bar: 200 µm.
(TIF)

## References

[1] Doyle KP, Simon RP, Stenzel-Poore MP (2008). Mechanisms of ischemic brain damage.. Neuropharmacology.

[2] Duncan P, Studenski S, Richards L, Gollub S, Lai SM (2003). Randomized clinical trial of therapeutic exercise in subacute stroke.. Stroke.

[3] Kwakkel G, van Peppen R, Wagenaar RC, Wood Dauphinee S, Richards C (2004). Effects of augmented exercise therapy time after stroke: a meta-analysis.. Stroke.

[4] Reynolds BA, Weiss S (1992). Generation of neurons and astrocytes from isolated cells of the adult mammalian central nervous system.. Science.

[5] Weiss S, Reynolds BA, Vescovi AL, Morshead C, Craig CG (1996). Is there a neural stem cell in the mammalian forebrain?. Trends Neurosci.

[6] Reynolds BA, Weiss S (1996). Clonal and population analyses demonstrate that an EGF-responsive mammalian embryonic CNS precursor is a stem cell.. Dev Biol.

[7] McKay R (1997). Stem cells in the central nervous system.. Science.

[8] Ming GL, Song H (2011). Adult neurogenesis in the mammalian brain: significant answers and significant questions.. Neuron.

[9] Lie DC, Song H, Colamarino SA, Ming GL, Gage FH (2004). Neurogenesis in the adult brain: new strategies for central nervous system diseases.. Annu Rev Pharmacol Toxicol.

[10] Johnson MA, Ables JL, Eisch AJ (2009). Cell-intrinsic signals that regulate adult neurogenesis in vivo: insights from inducible approaches.. BMB Rep.

[11] Khodosevich K, Monyer H (2010). Signaling involved in neurite outgrowth of postnatally born subventricular zone neurons in vitro.. BMC Neurosci.

[12] Imayoshi I, Sakamoto M, Yamaguchi M, Mori K, Kageyama R (2010). Essential roles of Notch signaling in maintenance of neural stem cells in developing and adult brains.. J Neurosci.

[13] Choi YS, Cho HY, Hoyt KR, Naegele JR, Obrietan K (2008). IGF-1 receptor-mediated ERK/MAPK signaling couples status epilepticus to progenitor cell proliferation in the subgranular layer of the dentate gyrus.. Glia.

[14] Bruel-Jungerman E, Veyrac A, Dufour F, Horwood J, Laroche S (2009). Inhibition of PI3K-Akt signaling blocks exercise-mediated enhancement of adult neurogenesis and synaptic plasticity in the dentate gyrus.. PLoS One.

[15] Leker RR, Lasri V, Chernoguz D (2009). Growth factors improve neurogenesis and outcome after focal cerebral ischemia.. J Neural Transm.

[16] Jacobs S, Lie DC, DeCicco KL, Shi Y, DeLuca LM (2006). Retinoic acid is required early during adult neurogenesis in the dentate gyrus.. Proc Natl Acad Sci U S A.

[17] Yabe T, Hirahara H, Harada N, Ito N, Nagai T (2010). Ferulic acid induces neural progenitor cell proliferation in vitro and in vivo.. Neuroscience.

[18] Heurteaux C, Gandin C, Borsotto M, Widmann C, Brau F (2010). Neuroprotective and neuroproliferative activities of NeuroAid (MLC601, MLC901), a Chinese medicine, in vitro and in vivo.. Neuropharmacology.

[19] Kim H (2005). Neuroprotective herbs for stroke therapy in traditional eastern medicine.. Neurol Res.

[20] Feigin VL (2007). Herbal medicine in stroke: does it have a future?. Stroke.

[21] Wu B, Liu M, Liu H, Li W, Tan S (2007). Meta-analysis of traditional Chinese patent medicine for ischemic stroke.. Stroke.

[22] Mook-Jung I, Hong HS, Boo JH, Lee KH, Yun SH (2001). Ginsenoside Rb1 and Rg1 improve spatial learning and increase hippocampal synaptophysin level in mice.. J Neurosci Res.

[23] Kim SJ, Son TG, Park HR, Park M, Kim MS (2008). Curcumin stimulates proliferation of embryonic neural progenitor cells and neurogenesis in the adult hippocampus.. J Biol Chem.

[24] Quintard H, Borsotto M, Veyssiere J, Gandin C, Labbal F (2011). MLC901, a traditional Chinese medicine protects the brain against global ischemia.. Neuropharmacology.

[25] Kim DH, Park SJ, Kim JM, Jeon SJ, Kim DH (2011). Cognitive dysfunctions induced by a cholinergic blockade and Aβ(25–35) peptide are attenuated by salvianolic acid B. Neuropharmacology.

[26] Tang MK, Ren DC, Zhang JT, Du GH (2002). Effect of salvianolic acids from Radix Salviae miltiorrhizae on regional cerebral blood flow and platelet aggregation in rats.. Phytomedicine.

[27] Chen T, Liu W, Chao X, Zhang L, Qu Y (2011). Salvianolic acid B attenuates brain damage and inflammation after traumatic brain injury in mice.. Brain Res Bull.

[28] Zhou L, Zuo Z, Chow MS (2005). Danshen: an overview of its chemistry, pharmacology, pharmacokinetics, and clinical use.. J Clin Pharmacol.

[29] Ji XY, Tan BK, Zhu YZ (2000). Salvia miltiorrhiza and ischemic diseases.. Acta Pharmacol Sin.

[30] Jiang M, Wang XY, Zhou WY, Li J, Wang J (2011). Cerebral protection of salvianolic acid A by the inhibition of granulocyte adherence.. Am J Chin Med.

[31] Tang M, Feng W, Zhang Y, Zhong J, Zhang J (2006). Salvianolic acid B improves motor function after cerebral ischemia in rats.. Behav Pharmacol.

[32] Du GH, Qiu Y, Zhang JT (2000). Salvianolic acid B protects the memory functions against transient cerebral ischemia in mice.. J Asian Nat Prod Res.

[33] Yonemori F, Yamada H, Yamaguchi T, Uemura A, Tamura A (1996). Spatial memory disturbance after focal cerebral ischemia in rats.. J Cereb Blood Flow Metab.

[34] Shioda N, Han F, Fukunaga K (2009). Role of Akt and ERK signaling in the neurogenesis following brain ischemia.. Int Rev Neurobiol.

[35] Chan CB, Liu X, Pradoldej S, Hao C, An J (2011). Phosphoinositide 3-kinase enhancer regulates neuronal dendritogenesis and survival in neocortex.. J Neurosci.

[36] Le Belle JE, Orozco NM, Paucar AA, Saxe JP, Mottahedeh J (2011). Proliferative neural stem cells have high endogenous ROS levels that regulate self-renewal and neurogenesis in a PI3K/Akt-dependant manner.. Cell Stem Cell.

[37] Nakatomi H, Kuriu T, Okabe S, Yamamoto S, Hatano O (2002). Regeneration of hippocampal pyramidal neurons after ischemic brain injury by recruitment of endogenous neural progenitors.. Cell.

[38] Wang C, Zhang M, Sun C, Cai Y, You Y (2011). Sustained increase in adult neurogenesis in the rat hippocampal dentate gyrus after transient brain ischemia.. Neurosci Lett.

[39] Yagita Y, Kitagawa K, Ohtsuki T, Takasawa Ki, Miyata T (2001). Neurogenesis by progenitor cells in the ischemic adult rat hippocampus.. Stroke.

[40] Liu J, Solway K, Messing RO, Sharp FR (1998). Increased neurogenesis in the dentate gyrus after transient global ischemia in gerbils.. J Neurosci 18: 7768.

[41] Takagi T, Nozaki K, Takahashi J, Yodoi J, Ishikawa M (1999). Proliferation of neuronal precursor cells in the dentate gyrus is accelerated after transient forebrain ischemia in mice.. Brain Res.

[42] Kee NJ, Preston E, Wojtowicz JM (2001). Enhanced neurogenesis after transient global ischemia in the dentate gyrus of the rat.. Exp Brain Res.

[43] Canales JJ (2010). Comparative neuroscience of stimulant-induced memory dysfunction: role for neurogenesis in the adult hippocampus.. Behav Pharmacol.

[44] Banta Lavenex P, Lavenex P (2009). Spatial memory and the monkey hippocampus: not all space is created equal.. Hippocampus.

[45] Xu M, Fu G, Qiao X, Wu WY, Guo H (2007). HPLC method for comparative study on tissue distribution in rat after oral administration of salvianolic acid B and phenolic acids from Salvia miltiorrhiza.. Biomed Chromatogr.

[46] Davis AA, Temple S (1994). A self-renewing multipotential stem cell in embryonic rat cerebral cortex.. Nature.

[47] Nitatori T, Sato N, Waguri S, Karasawa Y, Araki H (1995). Delayed neuronal death in the CA1 pyramidal cell layer of the gerbil hippocampus following transient ischemia is apoptosis.. J Neurosci.

[48] Pulsinelli WA, Brierley JB, Plum F (1982). Temporal profile of neuronal damage in a model of transient forebrain ischemia.. Ann Neurol.

[49] Morris R (1984). Developments of a water-maze procedure for studying spatial learning in the rat.. J Neurosci Methods.

