# Supplementary figures and images for: Direct Stimulation of Adult Neural Stem/Progenitor Cells In Vitro and Neurogenesis In Vivo by Salvianolic Acid B
Source: PLoS One. 2012 Apr 24;7(4):e35636. doi: 10.1371/journal.pone.0035636 (PMC3335811; doi:10.1371/journal.pone.0035636)

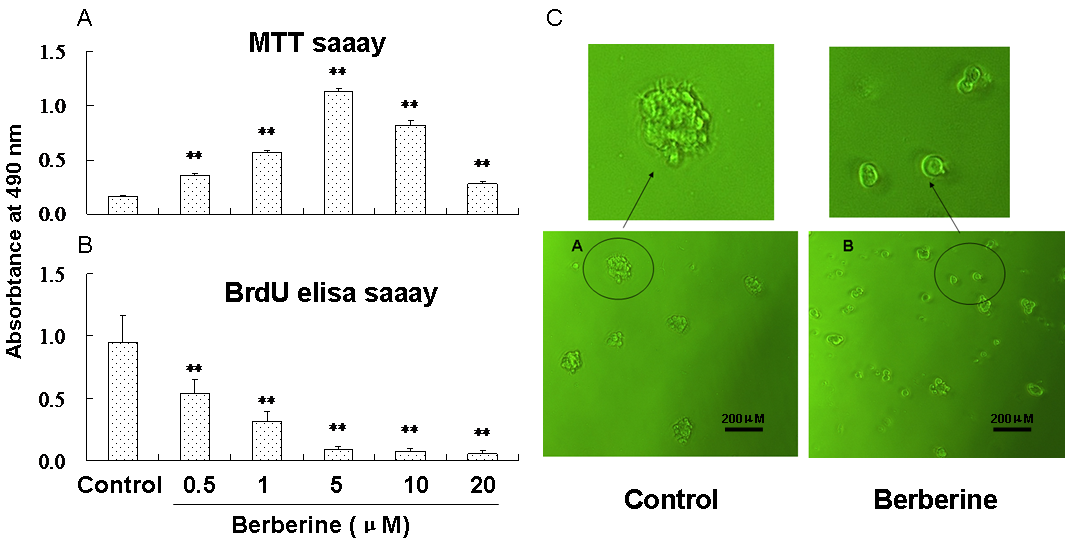

Supplement: Figure S1 — Berberine failed to promote the NSPCs proliferation. (A) Berberine increased the value of MTT assay. (B) Berberine reduced the BrdU incorporation. **Significant difference from the control group at P<0.01. (C) Morphologically berberine caused cell swelling and decreased the cell number. Scale bar: 200 µm. (TIF) [file pone.0035636.s001.tif]

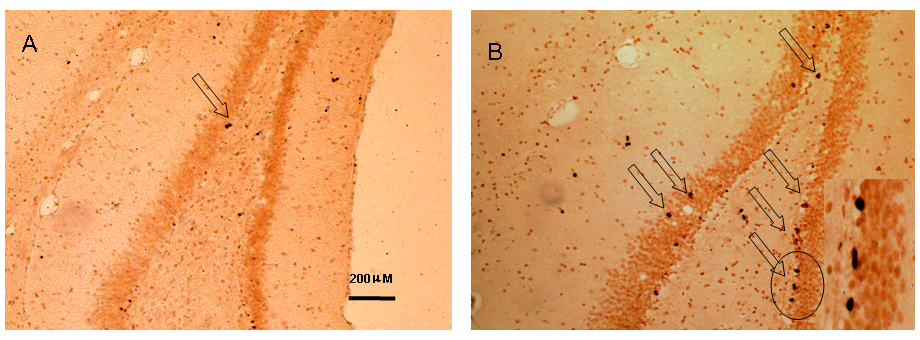

Supplement: Figure S2 — Regeneration of Hippocampal NSPCs Following Ischemia. BrdU positive cells in the granule cell layer in the intact (A) and ischemic (B, DAI7) animals. The inset shows an enlarged display of a typical BrdU positive cell. Scale bar: 200 µm. (TIF) [file pone.0035636.s002.tif]
